# Burden of *Mycobacterium ulcerans* Disease (Buruli Ulcer) and the Underreporting Ratio in the Territory of Songololo, Democratic Republic of Congo

**DOI:** 10.1371/journal.pntd.0002563

**Published:** 2013-12-05

**Authors:** Delphin Mavinga Phanzu, Patrick Suykerbuyk, Paul Saunderson, Philippe Ngwala Lukanu, Jean-Bedel Masamba Minuku, Désiré Bofunga B. Imposo, Blanchard Mbadu Diengidi, Makanzu Kayinua, Jean-Jacques Tamfum Muyembe, Pascal Tshindele Lutumba, Bouke C. de Jong, Françoise Portaels, Marleen Boelaert

**Affiliations:** 1 General Reference Hospital of Kimpese, Institut Médical Evangélique, Kimpese, Bas-Congo, Democratic Republic of Congo; 2 Department of Public Health, Unit of Epidemiology and Disease Control, Institute of Tropical Medicine, Antwerp, Belgium; 3 Department of Biomedical Sciences, Unit of Mycobacteriology, Institute of Tropical Medicine, Antwerp, Belgium; 4 American Leprosy Missions, Greenville, South Carolina, United States of America; 5 Central Office of the Rural Health Zone of Kimpese, Bas-Congo, Democratic Republic of Congo; 6 Central Office of the Rural Health Zone of Nsona Mpangu, Bas-Congo, Democratic Republic of Congo; 7 Institut National de Recherche Biomédicale, Kinshasa, Democratic Republic of Congo; Kwame Nkrumah University of Science and Technology (KNUST) School of Medical Sciences, Ghana

## Abstract

**Background:**

Cutaneous infection by *Mycobacterium ulcerans*, also known as Buruli ulcer (BU), represents the third most common mycobacterial disease in the world after tuberculosis and leprosy. Data on the burden of BU disease in the Democratic Republic of Congo are scanty. This study aimed to estimate the prevalence rate and the distribution of BU in the Songololo Territory, and to assess the coverage of the existing hospital-based reporting system.

**Methods:**

We conducted a cross-sectional survey (July–August 2008) using the door-to-door method simultaneously in the two rural health zones (RHZ) of the Songololo Territory (RHZ of Kimpese and Nsona-Mpangu), each containing twenty health areas. Cases were defined clinically as active BU and inactive BU in accordance with WHO-case definitions.

**Results:**

We detected 775 BU patients (259 active and 516 inactive) in a total population of 237,418 inhabitants. The overall prevalence of BU in Songololo Territory was 3.3/1000 inhabitants, varying from 0 to 27.5/1000 between health areas. Of the 259 patients with active BU, 18 (7%) had been reported in the hospital-based reporting system at Kimpese in the 6–8 months prior to the survey.

**Conclusion:**

The survey demonstrated a huge variation of prevalence between health areas in Songololo Territory and gross underreporting of BU cases in the hospital-based reporting system. Data obtained may contribute to better targeted and improved BU control interventions, and serve as a baseline for future assessments of the control program.

## Author Summary

Buruli ulcer (BU) is a necrotizing bacterial infection of skin, subcutaneous tissue and bone, caused by an environmental pathogen, *Mycobacterium ulcerans*. BU is considered as one of the Neglected Tropical Diseases with a poorly known global prevalence, and mainly affects remote rural African communities. Data on the burden of BU disease in the Democratic Republic of Congo (DRC) are scanty. The present study is the first exhaustive survey in DRC on the frequency of BU in the community. The survey demonstrated large variations in prevalence between health areas in Songololo Territory. Moreover, our data showed that the BU cases in the hospital-based reporting system reflect only the tip of the iceberg of the true active BU prevalence. Indeed, only one in thirteen active BU cases was notified at the hospital at Kimpese in the 6–8 months prior to the survey. The present data will serve as a baseline assessment for the evaluation of control interventions in the study area, and, more generally, this study aims to raise awareness about the issue of underdetection of BU and the importance of increasing access to diagnosis and care. As such, we hope the study will contribute to improved control of BU.

## Introduction

Cutaneous infection by *Mycobacterium ulcerans*, also known as Buruli ulcer (BU), represents the third most common mycobacterial disease in the world after tuberculosis and leprosy [1]. In Africa, children under 15 years old have the highest incidence, but healthy persons of all ages, races, and socioeconomic classes are susceptible [2], [3]. Rates of infection among males and females are equal [3]. BU most affects the extremities [2], [4], and is diagnosed in the majority of patients at the ulcerative stage [5]. The disease has a scattered focal distribution within endemic regions, which impedes accurate estimation of disease burden [5], [6].

BU is considered as one of the Neglected Tropical Diseases (NTDs) with a poorly known global prevalence [7], and mainly affects remote rural African communities [8]. A recent review on prevalence [9] reported that, of the estimated 7,000 cases of BU reported annually worldwide, more than 4,000 cases occur in Sub-Saharan Africa. The largest numbers of reported BU cases are from the West African countries of Côte d'Ivoire (about 2,000 cases annually), Benin and Ghana, each reporting about 1,000 cases a year [3]. Various prevalence rates (Table 1) have been reported from different endemic regions in Sub-Saharan Africa [6], [10]–[13].

**Table 1:** Prevalence of Buruli ulcer disease in Africa.

Year of report	Country	Study area	Overall prevalence active & inactive BU rate per 1000	Prevalence Active BU rate per 1000	Reference
2001	Ivory Coast	Nation-wide	-	0.3	Kanga and Kacou [10]
2002	Ghana	Nation-wide	0.31	0.21	Amofah and others [11]
		Amansie West District	-	1.51	
2004	Cameroon	Valley of Nyong river	4.42	2.05	Noeske and others [12]
2005	Benin	Lalo District	8.66	1.84	Johnson and others [6]
2009	Cameroon	Akonolinga District	4.70	2.50	Porten and others [13]

BU: Buruli ulcer.

In the Democratic Republic of Congo (DRC), more than 500 BU cases had been reported before 1980 [14]. The first BU case reports in the Province of Bas-Congo were published in the 1960s and 1970s [15]–[17]. However, in-depth interviews of former patients conducted in the Bas-Congo by Meyers et al. strongly supported the concept that BU was an ancient disease in that region [14]. After 1980, there was a silent period of 20 years without any cases reported in the scientific literature [14]. A national hospital-based survey conducted in 2004 identified 487 clinically suspected cases of BU from six provinces [18]. Between 2002–2004, an apparent resurgence of BU was reported in Songololo Territory [4], known to be the main focus of BU in the country [17]. Since the end of 2004, the General Reference Hospital (GRH) of the Institut Médical Evangélique (IME) of Kimpese launched a specialized BU program sponsored by American Leprosy Missions, offering in-patient treatment free-of-charge and supplementary aid. A recent study has shown a strong increase in the number of admitted BU cases at the IME Hospital after the start of the BU Control Project [19]. Although the number of BU cases admitted in the hospital was rising, data on the exact prevalence and the extent of the disease in the region was lacking. We set up a study to obtain relevant information for planning subsequent control activities, and to provide baseline data for future control program assessments. This study aimed (i) to assess the prevalence and the geographic distribution of BU, (ii) to determine the epidemiologic characteristics of BU, and (iii) to determine the project coverage in Songololo Territory, the target endemic region of the project.

## Methods

### Ethics statement

The Congolese Ministry of Health granted approval to conduct the survey. We obtained ethical clearance for this study from the Institutional Review Board of IME (N° IME/CS/01/2008). All patients, or their guardian in the case of minors, provided written informed consent for all diagnostic and treatment procedures and publication of any or all images derived from the management of the patient, including clinical photographs that might reveal patient identity. After informed consent had been given, data were recorded on a Community BU Form recommended by WHO. Patient care was free of charge.

### Survey zone

**Figure 1:**
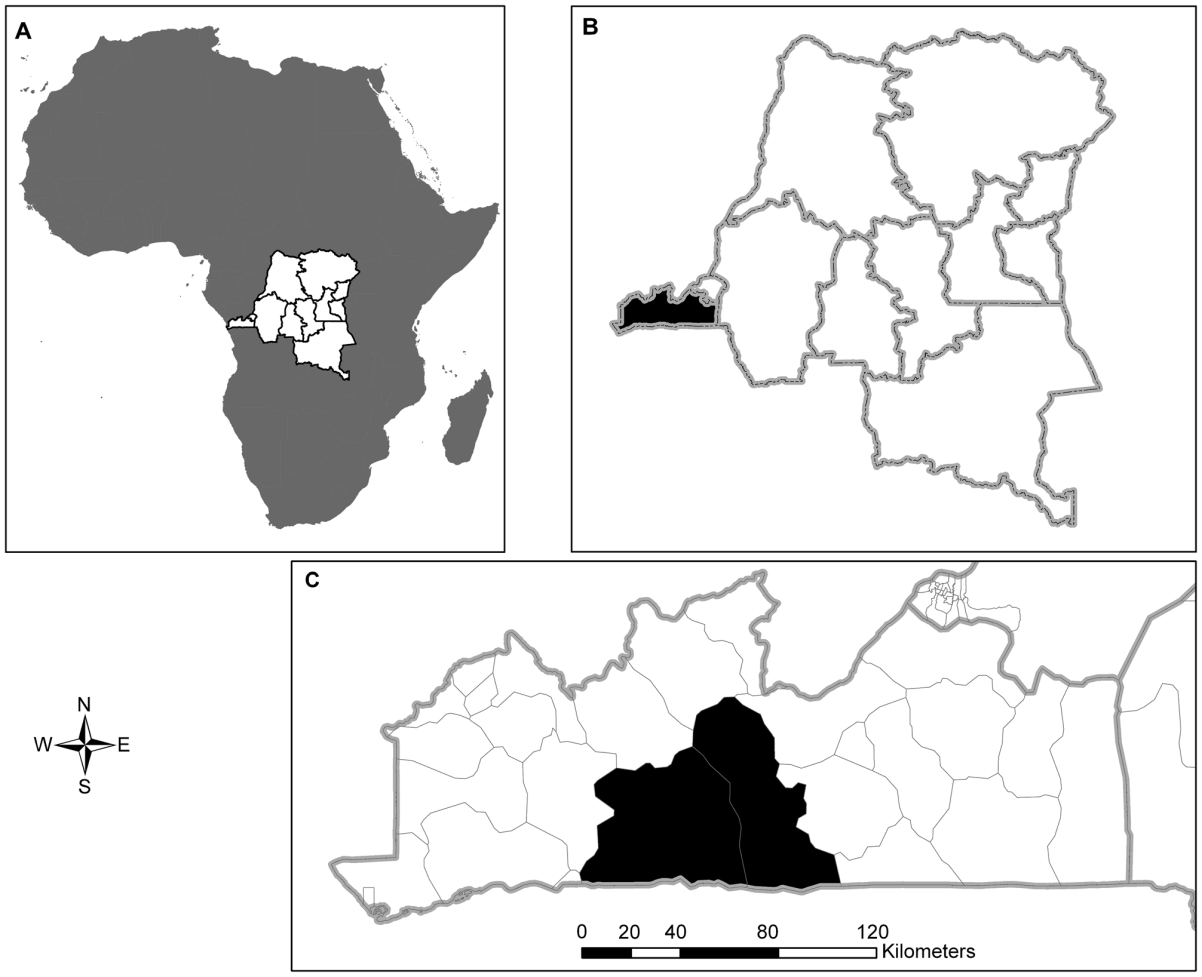
Location of the survey zone. A. Map of Africa showing the location of the Democratic Republic of Congo. B. Map of the Democratic Republic of Congo showing the location of the province of Bas-Congo. C. Map of the Province of Bas-Congo showing the location of the two health zones surveyed in 2008.

The case search covered two rural health zones (RHZ), Kimpese and Nsona-Mpangu, both located in Songololo Territory (Figure 1), one of ten territories of Bas-Congo Province. It is situated in the District of Cataractes and covers an area of 8,190 Km^2^, approximately 15.2% of the total surface of the province, with a population of 237,418 inhabitants in 2008 (enumeration conducted on December 2007 by the Central Offices of the 2 RHZ). An average of 6 persons per household was used as a regional estimate, giving a total of 39,569 households to be visited by 80 community health workers (CHW).

Songololo Territory is limited in the north by the Congo River, in the west by Sekebanza Territory, in the east by Mbanza-Ngungu Territory and in the south by the northern border of Angola. Each RHZ is subdivided into 20 health areas (Table S1 & Table S2). The primary level of health care facilities includes the Rural Health Posts (HP), Health Centres (HC) and Reference Health Centres (RHC), and the secondary level is represented by the GRH.

### Patients, data collection and analysis

We conducted a cross-sectional survey (July–August 2008) using the door-to-door method simultaneously in the two RHZ of the Songololo Territory (i.e., Kimpese and Nsona-Mpangu), each containing twenty health areas. Cases were defined clinically as active BU and inactive (healed) BU in accordance with WHO-case definitions [20]. We defined functional limitation as any reduction in the range of motion of one or more joints, and assessed it by clinical observation. Lesions were considered as mixed forms when the simultaneous presence of different forms of disease, including bone and joint involvement, in the same patient was noted. In addition, we defined as simple ulcerative forms (SUF) the ulcerative lesions not associated with other clinical lesions such as papule, nodule, plaque, edema or osteomyelitis at the same site. Lesions were categorized as follows: A single lesion <5 cm (Category I); a single lesion 5–15 cm (Category II); a single lesion >15 cm, multiple lesions, and lesions at critical sites (face, breast and genitalia) or osteomyelitis (Category III). The status of relapse was assessed by questioning the patients, or their guardian in the case of minors, on the history of the lesion, and defined as the reappearance of an ulcer or another form of the disease at the original site of the lesion or elsewhere during the 12 months that followed the end of the previous treatment (antibiotics and/or surgery).

This study was conducted in two phases: a preparatory phase and an investigation phase. During the preparatory four-week phase (June 2008), the purpose of the study was explained to the local political and health authorities, and their approval was obtained. Then, 80 CHW, i.e., 40 per RHZ, were trained in the use of the survey tools (BU community form, pictorial document to recognize BU) and in the identification of suspected BU cases in their communities. We also trained six physicians (working in the RHC of both RHZ), two nurse-supervisors of the leprosy and tuberculosis program (LT), and 40 head nurses (in charge of peripheral health areas), in active case-finding of BU cases and in the use of the survey tools.

For the survey, each RHZ was provided with 1 motor bike, 1 Global Position System device, 4 digital photo cameras, 30 bicycles (at least 1 for each health area), 25 megaphones (at least 1 for each health area), drugs and required medical and laboratory consumables.

The investigation phase was divided in two periods. The first period (two to three weeks depending on health area) consisted of making an inventory of all BU-like cases by the CHW, using the door-to-door approach in all villages and in each section of two cities in Songololo Territory (Songololo city and Kimpese city). The recommendation to CHW was to visit 40 households per day. A pictorial document, showing different clinical manifestations of BU, was presented to the head of the household or his/her representative asking if any household members presented similar lesions. If the head of the household was not present, the household was revisited once. The second period (6 weeks) included the clinical validation of suspected BU cases by trained health professionals. The eight validation teams were each composed of two people: firstly, a team member of the BU Project (physician or nurse), or another physician, or a LT supervisor, and secondly one of the head nurses.

The diagnostic confirmation process of suspected cases involved the collection of swabs from ulcerative lesions and fine needle aspirates from non-ulcerative lesions, followed by laboratory analyses (bacteriology and/or molecular biology) according to WHO recommendations [20]. The initial direct smear examinations for acid-fast bacilli were made at the IME/Kimpese laboratory, followed by in vitro culture for M. ulcerans. Samples were sent in tubes to the “Institut National de Recherche Biomédicale” in Kinshasa, DRC, where PCR for the detection of *M. ulcerans* DNA was performed, according to WHO recommendations [20]. The external quality control was conducted by the Unit of Mycobacteriology of the Institute of Tropical Medicine in Antwerp, Belgium.

The study was carried out simultaneously in the different health areas of both RHZ. Data were recorded on a standardized Case Registry Form elaborated by WHO (BU02), entered into an Excel database (Microsoft Corporation, Redmond, WA) and analyzed with Epi-Info version 3.3.2 (Centers for Diseases Control and Prevention, Atlanta, GA). The Pearson chi-square test was used to compare proportions with a significance level set at 5%, and the Fisher's exact test when an expected cell value was less than 5. Coverage was calculated as the number of active cases detected who had visited the BU reference center in IME Hospital. We produced the distribution maps of BU in Songololo Territory using ArcGIS 9.2 (ESRI, Redlands, CA, USA).

## Results

**Figure 2:**
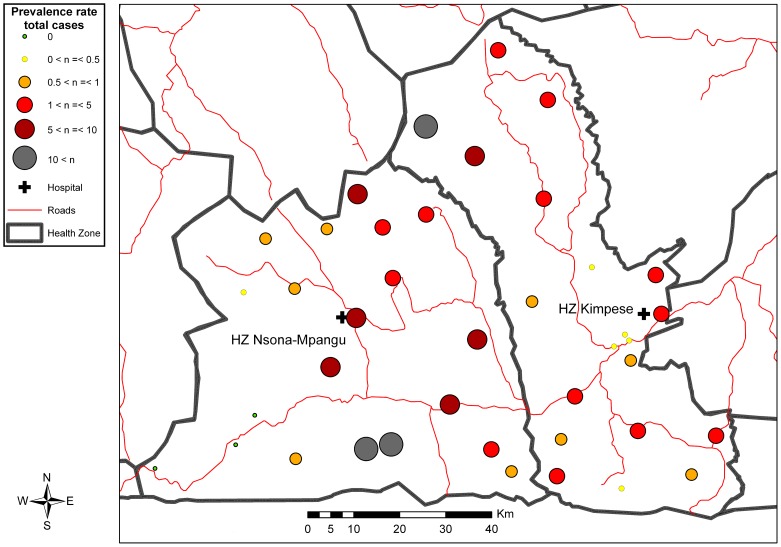
Distribution of total number of BU cases (active and inactive) in the Songololo Territory, July–August 2008.

**Figure 3:**
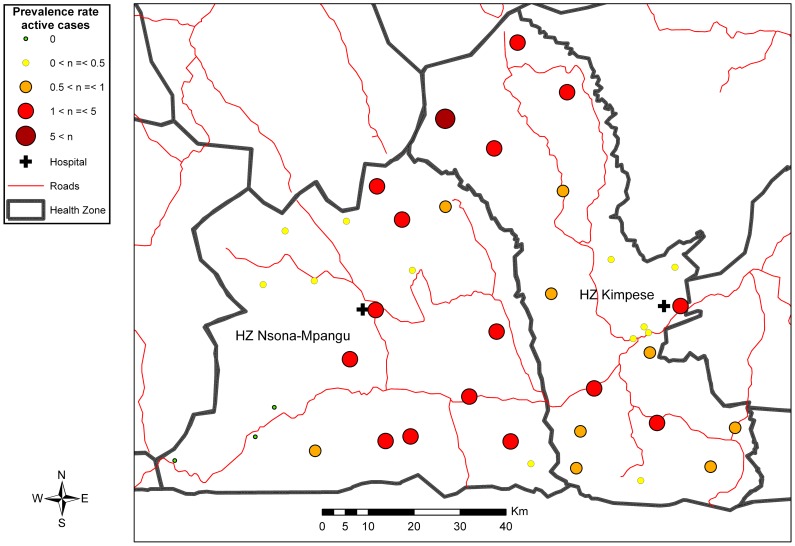
Distribution of active BU cases in the Songololo Territory, July–August 2008.

The CHW visited a total of 39,044 households distributed across 9 sections of two cities (Kimpese and Songololo), 46 hamlets and camps, and 547 villages of the Songololo Territory. The estimated coverage of the study was 98.6%. During the household visits, the CHW inventoried 2,516 persons with BU-like lesions, among which 775 (30.8%) were validated in a second step as probable cases of BU, all forms included (i.e., 259 with active and 516 with inactive lesions). A total of 72 out of 241 (30%) patients with active lesions in whom a sample could be taken were confirmed by at least one positive laboratory test for *M. ulcerans*. The overall prevalence of BU (active and inactive) in Songololo Territory was 3.3/1000 inhabitants, varying from 0 to 27.5/1000 between health areas, while the prevalence of active BU was 1.1/1000 inhabitants with the minimum of 0.3/1000 when only active, laboratory confirmed BU, was considered. Table 2 shows the prevalence of different BU forms in both RHZ of Songololo Territory, and the distribution per health area is presented in Figures 2, 3, S1 and S2. The overall prevalence for the RHZ of Kimpese was 2.6 per 1000 inhabitants and could vary between health areas from 0.1 (Kimbanguiste) to 24.4 (Mukimbungu). The prevalence of BU active forms was 1 per 1000 inhabitants, varying between health areas from 0.1 (Kimbanguiste) to 5.7 (Mukimbungu). The health areas of Mukimbungu and Kasi, located in the North of the RHZ of Kimpese, are the most endemic, representing together 60% of the identified patients during the survey (Table S1).

**Table 2:** Prevalence of Buruli ulcer in the Territory of Songololo, July–August 2008.

Health Zone	Population	Number BU case Total	Number Active BU	Number Inactive BU	Global prevalence/10^3^	Active BU prevalence/10^3^	Inactive BU prevalence/10^3^
Kimpese	146,108	376	141	235	2.6	1	1.6
Nsona-Mpangu	91,310	399	118	281	4.4	1.3	3.1
Total	237,418	775	259	516	3.3	1.1	2.2

BU: Buruli ulcer.

Sixty percent of the identified patients in the RHZ of Nsona-Mpangu were from 3 health areas, Kisonga, Nkamuna, and Songololo (Table S2). The overall prevalence in this RHZ was 4.4 per 1000 inhabitants, varying from 0 (health areas Nduizi, Nkenge and Pala Bala) to 27.5 (Kisonga). The prevalence of active forms of BU was 1.3 per 1000 inhabitants, varying between health areas from 0 (Nduizi, Nkenge and Pala Bala) to 3.8 (Kisonga).

The age distribution of all cases ranged from 2 to 94 years (Median 27, Interquartile range (IQR) 14–44) with no significant differences between active and inactive cases. The supplementary tables provide the detailed information.

We observed a predominance of female gender (60%) among the recorded cases. Among the 259 patients with active lesions, no sex difference was observed, as 130 (50.2%) were female. The proportion of new cases was far higher (94%) than the relapses. The ages ranged from 2 to 94 years (Median 27 years; IQR 11–47 years), and the distributions in the two RHZ were similar. Among these 259 patients, 192 (74%) had ulcerative lesions and 62 (23.9%) were diagnosed with functional joint limitations. Lesions on the limbs were predominant, representing 90% of the sites of lesions. Regarding the patients' categorization, 48.8% were in category I, 31.5% category II, and 19.7% category III. The proportion of patients with ulcerative lesions was higher (p<0.001) in the RHZ of Kimpese (83%) compared to the RHZ Nsona-Mpangu (63.6%). Less than half of the patients of the RHZ of Kimpese (41.2%) and more than half (57.6%) in the RHZ of Nsona-Mpangu were in category I (p = 0.031) (Table S3).

Female patients predominated amongst active confirmed cases compared to unconfirmed cases; on the other hand, male patients were more frequent in active unconfirmed patients (p = 0.029). No differences in the age distribution were observed between active confirmed and unconfirmed patients. The lower limb locations were significantly more frequent amongst active unconfirmed patients (p<0.001). Upper limb sites predominated (p<0.001) amongst active confirmed patients (Table 3).

**Table 3:** Clinico-epidemiological features of active BU cases in the Territory of Songololo, July–August 2008.

Characteristic		Active confirmed (n = 72)	Active unconfirmed (n = 187)	p-value*
		n (%)	n (%)	
Gender	Female	44 (61.1)	86 (46.0)	0.029
	Male	28 (38.9)	101 (54.0)	
Age	≤15 years	27 (37.5)	61 (32.6)	0.298
	16–49 years	35 (48.6)	84 (44.9)	
	>49 years	10 (13.9)	42 (22.5)	
Classification of cases	New case	64 (88.9)	179 (95.7)	0.078†
	Relapse	8 (11.1)	8 (4.3)	
Clinical forms				
	Ulcerated simple	55 (76.4)	123 (65.8)	0.200
	Ulcerated mixed	4 (5.6)	10 (5.3)	
	Non ulcerated	13 (18.0)	54 (28.9)	
Category of lesion	I	36 (50.0)	88 (48.4)‡	0.740
	II	24 (33.3)	56 (30.8)‡	
	III	12 (16.7)	38 (20.9)‡	
Functional limitation	Yes	22 (30.6)	40 (21.4)	0.121
	No	50 (69.4)	147 (78.6)	
Site of lesion	Lower limb	39 (53.4)Ω	136 (72.7)	<0.001
	Upper limb	29 (39.7)Ω	31 (16.6)	
	Other	5 (6.8)Ω	20 (10.7)	
Rural Health Zone	Kimpese	41 (56.9)	100 (53.5)	0.615
	Nsona Mpangu	31 (43.1)	87 (46.5)	

* X^2^ test unless otherwise specified.

†Two-sided Fisher exact test (An expected cell value was less than 5).

‡n = 182 because of 5 missing data.

Ωn = 73 because of one case with disseminated lesions.

Features of active cases in the two RHZ were quite similar, with a few exceptions. The ulcerated forms (p<0.001) and functional limitations on diagnosis (p<0.001) predominated in the RHZ of Kimpese. Features of inactive cases in the two RHZ were similar but functional limitations were more often observed in the RHZ of Kimpese (p = 0.005) (Table S4).

Only 25 BU patients were admitted and notified at the General Hospital IME/Kimpese between January and August 2008, amongst which 18 were still under treatment for active BU during the survey. Thus, 93% of all active BU patients at the time of the community survey were not captured by the hospital-based reporting system, corresponding to a ratio of 1 reported case for approximately 13 unreported cases.

## Discussion

The present study is the first exhaustive population-based survey in DRC aiming to assess the prevalence and distribution of BU in a well-circumscribed endemic region. The survey demonstrated a huge variation in prevalence between health areas and gross underreporting of BU cases in Songololo Territory, compared with the ongoing hospital-based reporting system.

Case-definition during the survey was essentially clinical. Case validation was performed by physicians from the BU project and physicians working in the area, well-trained in BU diagnosis, assisted by either a nurse from the BU project or a LT-supervisor, with the nurse responsible for the health area. We are aware of the limitations of clinical diagnosis, which is dependent on the range of experience of health professionals. This may account for certain non-BU cases included in this study. In endemic regions, depending on the clinical stage of the disease, BU may be confused with many other conditions such as nodular onchocerciasis, cyst, lipoma, lymphadenitis, phagedenic tropical ulcer, pyomyositis, necrotizing fasciitis [20], [21], to name a few. Our study showed that 72 out of the 241 (30%) patients who were tested, were confirmed in the laboratory. The low confirmation rate is mostly due to the relatively high number (almost half) of the ulcers being in an advanced stage of healing. Likewise, the technical problems encountered by peripheral health professionals when sampling non-ulcerated lesions and wounds, where mixtures of traditional herbs had been applied, may have played a role. Nevertheless, lesions due to another etiology misclassified as BU cannot be excluded, as lower limb locations were significantly more frequent among active unconfirmed patients. Indeed, among 92 clinically suspected patients recruited from the RHZ of Nsona Mpangu, Kibadi et al. found 31 (33.7%) PCR negative patients and among them, 25 with histopathological features not compatible with BU (chronic inflammation and bacterial infections due to gram positive cocci) [22].

Despite these limitations, we suggest that our results reflect the endemicity of BU in Songololo Territory reasonably well. In fact, the areas previously established as most endemic were corroborated through this survey, as were the non- or hypoendemic areas [15]–[17], [4].

When considering only active lesions, no sex difference was observed, similar to findings in other studies [2], [11], [12], [23], [24], although our study showed a predominance of females among all cases detected (active and inactive), because among inactive cases, 64.9% (335/516) were females and only 35.1% (181/516) were males. Females predominated also among active confirmed BU cases. This preponderance may be due to time itself, or the fact that the population was predominantly female. When referring to the national census figures (July 2008 estimates), for a total population of 66,514,504 inhabitants, 50.3% were female and 49.7% male.

Among the 259 patients with active lesions, the majority (66%) were over age 15, similar to previous findings in the same area [19]. Ages observed in this survey were higher than found in other disease-endemic countries [2], [10], [12], [25]. The median ages for both RHZ were similar with the median age of 25 years found in Ghana [11], and relatively high when compared to the 15.5 years observed in Cameroon [13]. The predominant clinical presentation was an ulcerative lesion in 192 cases (74%). This is consistent with studies in Côte d'Ivoire [10] and Cameroon [12], [13], while the percentage of ulcerative lesions was lower in some other studies, for example, 48.5% in Ghana [11], approximately 50% in Benin from 1997 to 2001 [26] and 57.5% in 2004 in the same country [6]. Of the 259 active cases, 62 (23.9%) were diagnosed with joint functional limitations, similar to previous findings in the same area [19], and in other African endemic regions [6], [12]. The general finding of limbs being most affected was confirmed in this study [2], [11]–[13], [23], [24].

The results presented in Table 3 shows that nearly 50% of the BU patients had category I lesions. A similar observation was made in the District of Akonolinga, Cameroon [13]. Ambulatory treatment, based on antibiotic therapy in the primary health care facility, is indicated for this category of patients. Indeed, most category I and some category II lesions may heal completely with antibiotic treatment alone [3], [27]. The introduction of antibiotic therapy [28] has shifted the balance between surgical treatment, mainly limited to reference centers, and antibiotics administered at the most peripheral level of the health system [3].

The clinical presentation of BU was different in the two health zones (Table S3). The degree of functional limitation was significantly higher in patients in Kimpese and they had more often ulcerated lesions. We speculate that this difference is most likely due to differences in health seeking behavior, with higher patient delays in Kimpese, notwithstanding the fact that they were living at shorter distance from the IME hospital. In recent years, an influential religious sect has been a factor in the reluctance to seek medical care in the Kimpese area.

Although the number of BU patients admitted at the hospital has increased in recent years, the survey results have demonstrated that the coverage of the population at risk was still insufficient. Of the 259 patients with active BU, 18 (7%) had been reported in the hospital-based reporting system. Porten et al. reported a coverage of 16%, limited to the area close to the Akonolinga hospital in Cameroon, where Médecins Sans Frontières (MSF) opened a BU programme in 2002. The need for improved access to care in the high prevalence areas was emphasized [13]. In the same area, Grietens et al. found that despite the significant reduction in costs for medical care, hospital treatment for BU often remained financially and socially unaffordable for patients and their households, leading to the abandonment of biomedical treatment, or avoiding it altogether. They concluded in their study that from a socio-economic perspective, a decentralized treatment system may limit the impoverishment of households caused by a long hospitalization period [29]. We agree with this opinion because bringing treatment as close as possible to the communities will have a significant mitigating impact on the socio-economic repercussions of BU.

The survey demonstrated large variations in prevalence between health areas within an endemic health zone consistent with previous studies in other African BU-endemic regions [6], [12], [13].

Tables S1 and S2 show that in both RHZ, 60% of patients were respectively identified from 2 out of 20 health areas (Mukimbungu, Kasi) in the RHZ of Kimpese and 3 out of 20 health areas (Kisonga, Nkamuna, Songololo) in the RHZ of Nsona-Mpangu.

Therefore, priority in case detection should be given to the most endemic health areas. A close collaboration with the provincial Leprosy & Tuberculosis control officers may facilitate the integration of BU activities at the primary health care centers. In fact, the use of the same case-confirmation network or the organization of integrated supervisions would help to reduce the BU intervention costs.

Data obtained in this survey may contribute to better targeted and improved BU control interventions, and serve as a baseline for future assessments of the control program.

## Supporting Information

Checklist S1 STROBE Checklist.
(DOC)

Figure S1 Distribution of confirmed active BU cases in the Songololo Territory, July–August 2008.
(TIF)

Figure S2 Distribution of inactive BU cases in the Songololo Territory, July–August 2008.
(TIF)

Table S1 Distribution of active and inactive BU cases in the Rural Health Zone of Kimpese (July–August 2008).
(DOCX)

Table S2 Distribution of active and inactive BU cases in the Rural Health Zone of Nsona Mpangu (July–August 2008).
(DOCX)

Table S3 Comparison of active case features in the two Rural Health Zones of Songololo Territory, July–August 2008.
(DOCX)

Table S4 Comparison of inactive case features in the two Rural Health Zones of Songololo Territory, July–August 2008.
(DOCX)
