# Supplementary material for: Burden of Mycobacterium ulcerans Disease (Buruli Ulcer) and the Underreporting Ratio in the Territory of Songololo, Democratic Republic of Congo
Source: PLoS Negl Trop Dis. 2013 Dec 5;7(12):e2563. doi: 10.1371/journal.pntd.0002563 (PMC3855042; doi:10.1371/journal.pntd.0002563)
Supplement: Checklist S1 — STROBE Checklist. (DOC) [file pntd.0002563.s001.doc]

STROBE Statement—Checklist of items that should be included in reports of ***cross-sectional studies***

|  | Item No | Recommendation |
| --- | --- | --- |
| **Title and abstract** | 1 | (*a*) Indicate the study’s design with a commonly used term in the title or the abstract  Cross-sectional study |
|  |  | (*b*) Provide in the abstract an informative and balanced summary of what was done and what was found  We conducted a two-month (July-August 2008) cross-sectional survey using the door-to-door method simultaneously in the two rural health zones (RHZ) of the Songololo Territory (RHZ of Kimpese and Nsona-Mpangu), each containing twenty health areas. Cases were defined clinically as active BU and inactive BU in accordance with WHO-case definitions.  We detected 775 BU patients (259 active and 516 inactive) in a total population of 237,418 inhabitants. The overall prevalence of BU in Songololo Territory was 3.3/1000 inhabitants, varying from 0 to 27.5/1000 between health areas. Of the 259 patients with active BU, 18 (7%) had been reported in the hospital-based reporting system at Kimpese in the 6-8 months prior to the survey. |
| Introduction | | |
| Background/rationale | 2 | Explain the scientific background and rationale for the investigation being reported  A recent study has shown a strong increase in the number of admitted BU cases at the IME Hospital after the start of the BU Control Project (19). Although the number of BU cases admitted in the hospital was rising, data on the exact prevalence and the extent of the disease in the region was lacking. We set up a study to obtain relevant information for control activities planning, and to provide baseline data for future control program assessments. |
| Objectives | 3 | State specific objectives, including any pre specified hypotheses  This study aimed (i) to assess the prevalence and the geographic distribution of BU, (ii) to determine the epidemiologic characteristics of BU, and (iii) to determine the project coverage in Songololo Territory, the target endemic region of the project. |
| Methods | | |
| Study design | 4 | Present key elements of study design early in the paper  We conducted a two-month (July-August 2008) cross-sectional survey using the door to door method simultaneously in the two RHZ of the Songololo Territory (i.e., Kimpese and Nsona-Mpangu), containing each twenty health areas. |
| Setting | 5 | Describe the setting, locations, and relevant dates, including periods of recruitment, exposure, follow-up, and data collection  The case search covered two rural health zones (RHZ), Kimpese and Nsona-Mpangu, both located in Songololo Territory (Figure 1), one of ten territories of Bas-Congo Province. It is situated in the District of Cataractes and covers an area of 8,190 Km2, approximately 15,2% of the total surface of the province, with a population of 237,418 inhabitants in 2008 (Estimation from the Central Offices of the 2 RHZ).  Songololo Territory is limited in the north by the Congo River, in the west by Sekebanza Territory, in the east by Mbanza-Ngungu Territory and in the south by the northern boundary of Angola. Each RHZ is subdivided into 20 health areas (Table S1 & Table S2, Figure 1). The primary level of health care facilities includes the Rural Health Posts (HP), Health Centres (HC) and Reference Health Centres (RHC), and the secondary level is represented by the GRH.  The study was carried out simultaneously in the different health area of both RHZ. Data were recorded in a standardized Case Registry Form elaborated by WHO (BU02), entered into an Excel database (Microsoft Corporation, Redmond, WA) and analyzed with Epi-Info version 3.3.2 (Centers for Diseases Control and Prevention, Atlanta, GA). |
| Participants | 6 | (*a*) Give the eligibility criteria, and the sources and methods of selection of participants  Cases were defined clinically as active BU and inactive (healed) BU in accordance with WHO-case definitions (20). |
| Variables | 7 | Clearly define all outcomes, exposures, predictors, potential confounders, and effect modifiers. Give diagnostic criteria, if applicable  Cases were defined clinically as active BU and inactive (healed) BU in accordance with WHO-case definitions (20).  Functional limitation was defined as any reduction in the range of motion of one or more joints, and was assessed based on clinical observation.  Lesions were considered as mixed forms when simultaneous presence of different forms of disease including bone and joint involvement in the same patient was noticed. In addition, we defined as simple ulcerative forms (SUF) the ulcerative lesions not associated with other clinical lesions such as papule, nodule, plaque, edema or osteomyelitis at the same site.  Lesions were categorized as follows: A single lesion < 5 cm (Category I); a single lesion 5-15 cm (Category II); a single lesion > 15 cm, multiple lesions, lesions at critical sites (face, breast and genitalia) or osteomyelitis (Category III). |
| Data sources/ measurement | 8* | For each variable of interest, give sources of data and details of methods of assessment (measurement). Describe comparability of assessment methods if there is more than one group  Data were recorded in a standardized Case Registry Form elaborated by WHO (BU02), entered into an Excel database (Microsoft Corporation, Redmond, WA) and analyzed with Epi-Info version 3.3.2 (Centers for Diseases Control and Prevention, Atlanta, GA). |
| Bias | 9 | Describe any efforts to address potential sources of bias  The investigation phase was divided in two periods.  The first period (two to three weeks depending on health area) consisted of making an inventory of all BU-like cases by the CHW, using the door-to-door approach in all villages and the quarters of two cities in Songololo Territory (Songololo city and Kimpese city).  The second period (6 weeks) included the clinical validation of suspected BU cases by trained health professionals. The eight validation teams were composed of either a team member of BU Project (physician or nurse), either a peripheral physician, or a LT supervisor, and a head nurse. |
| Study size | 10 | Explain how the study size was arrived at  The present study is the first exhaustive survey in DRC on the frequency of BU in the community. The investigation phase was divided in two periods. The first period (two to three weeks depending on health area) consisted of making an inventory of all BU-like cases by the CHW, using the door-to-door approach in all villages and the quarters of two cities in Songololo Territory (Songololo city and Kimpese city). An average of 6 persons per household was used as an estimate, giving a total of 39,569 households to be visited by 80 CHW. So, the recommendation to CHW was to visit 40 households per day. The pictorial document was shown to the head of the household or his/her representative asking if any household members presented similar lesions. If the head of the household was not present, the household was revisited once. The second period (6 weeks) included the clinical validation of suspected BU cases by trained health professionals. The eight validation teams were composed of either a team member of BU Project (physician or nurse), either a peripheral physician, or a LT supervisor, and a head nurse. |
| Quantitative variables | 11 | Explain how quantitative variables were handled in the analyses. If applicable, describe which groupings were chosen and why |
| Statistical methods | 12 | (*a*) Describe all statistical methods, including those used to control for confounding  The Pearson chi-square test was used to compare proportions with a significance level set at 5%, and the Fisher’s exact test when an expected cell value was less than 5. Coverage was calculated as the number of detected active cases who had visited the BU center of reference in IME Hospital. We produced the distribution map of BU in Songololo Territory using ArcGIS 9.2 (ESRI, Redlands, CA, USA). |
|  |  | (*b*) Describe any methods used to examine subgroups and interactions |
|  |  | (*c*) Explain how missing data were addressed |
|  |  | (*d*) If applicable, describe analytical methods taking account of sampling strategy |
|  |  | (*e*) Describe any sensitivity analyses |
| Results | | |
| Participants | 13* | (a) Report numbers of individuals at each stage of study—eg numbers potentially eligible, examined for eligibility, confirmed eligible, included in the study, completing follow-up, and analysed  During the household visits conducted in July 2008, the CHW inventoried 2516 persons with BU-like lesions, among which 775 (30.8%) were in a second step validated as probable cases of BU, all forms included (i.e., 259 with active and 516 inactive lesions). |
|  |  | (b) Give reasons for non-participation at each stage |
|  |  | (c) Consider use of a flow diagram |
| Descriptive data | 14* | (a) Give characteristics of study participants (eg demographic, clinical, social) and information on exposures and potential confounders  Details of the clinical characteristics are shown in table 3. |
|  |  | (b) Indicate number of participants with missing data for each variable of interest  5 missing data for the category of lesions (Table 3). |
| Outcome data | 15* | Report numbers of outcome events or summary measures  The overall prevalence of BU in Songololo Territory was 3.3/1000 inhabitants, varying from 0 to 27.5/1000 between health areas. Table 2 shows the prevalence of different BU forms in both RHZ of Songololo Territory, and the distribution per health area are presented in figures 2 and 3. The prevalence rates (square root transformed) of all health areas are shown in figures 2 and 3. The distribution map of BU by health area in both RHZ of Songololo Territory is shown in Figure 1.  We observed a predominance of female gender (60%) among the recorded cases, independently of the clinical forms (active or inactive). However, among the 259 patients with active lesions, no difference was observed between sexes. Indeed 130 (50.2%) were female. The proportion of new cases was far higher (94%) than the relapses. The ages ranged from 2 to 94 years (Median 27.5 years; Interquartile range (IQR) 14-46.5 years in the RHZ of Kimpese, and median 26 years; IQR 14-42 years in the RHZ of Nsona-Mpangu). Among these 259 patients, 88 (34%) were below 15 years old, 192 (74%) had ulcerative lesions and 62 (23.9%) were diagnosed with joint functional limitations. Lesions on the limbs were predominant, representing 90% of the sites of lesions. Regarding the patients’ categorization, 48.8% were from category I, 31.5% category II, and 19.7% category III. The proportion of patients with ulcerative lesions was higher (p= <0.001) in the RHZ of Kimpese (83%) compared to the RHZ Nsona-Mpangu (63.6%). Less than half of the patients of the RHZ of Kimpese (41.2%) and more than half (57.6%) in the RHZ of Nsona-Mpangu were from category I. The proportion of patients with joint functional limitations at diagnosis was higher (p= <0.001) in the RHZ Kimpese (17.8%) compared to the RHZ of Nsona-Mpangu (4.5%). Details of the clinical characteristics are shown in table 3.  Only 25 BU patients were admitted and notified at the General Hospital IME/Kimpese between January and August 2008, amongst which 18 were still under treatment for active BU during the survey. Thus, 93% of all active BU patients at the time of the community survey were not captured by the hospital-based reporting system, corresponding to a ratio of 1 reported case for approximately 13 unreported cases. |
| Main results | 16 | (*a*) Give unadjusted estimates and, if applicable, confounder-adjusted estimates and their precision (eg, 95% confidence interval). Make clear which confounders were adjusted for and why they were included |
|  |  | (*b*) Report category boundaries when continuous variables were categorized |
|  |  | (*c*) If relevant, consider translating estimates of relative risk into absolute risk for a meaningful time period |
| Other analyses | 17 | Report other analyses done—eg analyses of subgroups and interactions, and sensitivity analyses |
| Discussion | | |
| Key results | 18 | Summarise key results with reference to study objectives  The overall prevalence of all BU cases in Songololo Territory was 3.3 per 1000 inhabitants, varying from 0 to 27.5 per 1000 between health areas. The survey demonstrated large variations in prevalence between health areas within an endemic health zone consistent with previous studies in other African BU-endemic regions (6,12,13). When only active lesions are considered the prevalence in Songololo was 1.1 per 1000 inhabitants.  The results presented in table 3 shows that nearly 50% of the BU patients had category I lesions. The survey showed that lesions of categories I and II represented 80.3% of BU patients and 19.7% had been of category III, compared to respectively 55% and 45% among patients who presented in the hospital at Kimpese in the 6-8 months prior to the survey. When considering only active lesions, no sex difference was observed, similar to findings in other studies (2,11,12, ,23,24). However, our study showed a predominance of female gender among all cases detected (active and inactive) because among inactive cases, 64.9% (335/516) were females and only 35.1% (181/516) were males (data not shown). Among these 259 patients with active lesions, the majority (66%) were over age 15, similar to previous findings in the same area (19). The predominant clinical presentation was an ulcerative lesion in192 cases (74%). Of the 259 active cases, 62 (23.9%) were diagnosed with joint functional limitations, similar with previous findings in the same area (19), and in other African endemic regions (6,12).).  Although the number of BU patients admitted at the hospital increased over the last years, the survey results have demonstrated that the coverage of the population at risk was still insufficient. Of the 259 patients with active BU, 18 (7%) had been reported in the hospital-based reporting system. |
| Limitations | 19 | Discuss limitations of the study, taking into account sources of potential bias or imprecision. Discuss both direction and magnitude of any potential bias  Case-definition during the survey was essentially clinical. According to the WHO, clinical diagnosis is reliable in endemic regions if performed by an experienced health professional (20). In our study, case validation was performed by physicians of the BU project and physicians working at the periphery, well-trained in BU diagnosis, assisted by either a nurse of the BU project or a LT-supervisor with the nurse responsible for the health area. However, we are aware of the limitations of clinical diagnosis, which is dependent on the range of the experience of health professionals. This may account for certain non-BU cases included in this study. Our study showed that 72 out of 241 (30%) patients with sampling done were laboratory confirmed. The low confirmation rate is mostly due to the relatively high number (almost the half) of ulcers in advanced healing stage, and technical problems encountered by peripheral health professionals when sampling non-ulcerated lesions and wounds where mixture of traditional herbs had been applied, although lesions due to another etiology misclassified as BU cannot be excluded. |
| Interpretation | 20 | Give a cautious overall interpretation of results considering objectives, limitations, multiplicity of analyses, results from similar studies, and other relevant evidence  However, we assume that our results reflect well the endemicity of BU in Songololo Territory. In fact, the previously established most endemic areas were confirmed through this survey, as well as the non- or hypoendemic areas (15-17,4). |
| Generalisability | 21 | Discuss the generalisability (external validity) of the study results |
| Other information | | |
| Funding | 22 | Give the source of funding and the role of the funders for the present study and, if applicable, for the original study on which the present article is based  This study was supported by the American Leprosy Missions (Greenville, South Carolina, United States of America), the European Commission  (International Science and Technology Cooperation Development Program), Project No. INCO-CT-2005-05-051476-BURULICO and the Directorate General for Development and Cooperation (Brussels, Belgium). The funders had no role in study design, data collection and analysis, decision to publish, or preparation of the manuscript. |

*Give information separately for exposed and unexposed groups.

**Note:** An Explanation and Elaboration article discusses each checklist item and gives methodological background and published examples of transparent reporting. The STROBE checklist is best used in conjunction with this article (freely available on the Web sites of PLoS Medicine at http://www.plosmedicine.org/, Annals of Internal Medicine at http://www.annals.org/, and Epidemiology at http://www.epidem.com/). Information on the STROBE Initiative is available at www.strobe-statement.org.
