# Supplementary figures and images for: Burden of Mycobacterium ulcerans Disease (Buruli Ulcer) and the Underreporting Ratio in the Territory of Songololo, Democratic Republic of Congo
Source: PLoS Negl Trop Dis. 2013 Dec 5;7(12):e2563. doi: 10.1371/journal.pntd.0002563 (PMC3855042; doi:10.1371/journal.pntd.0002563)

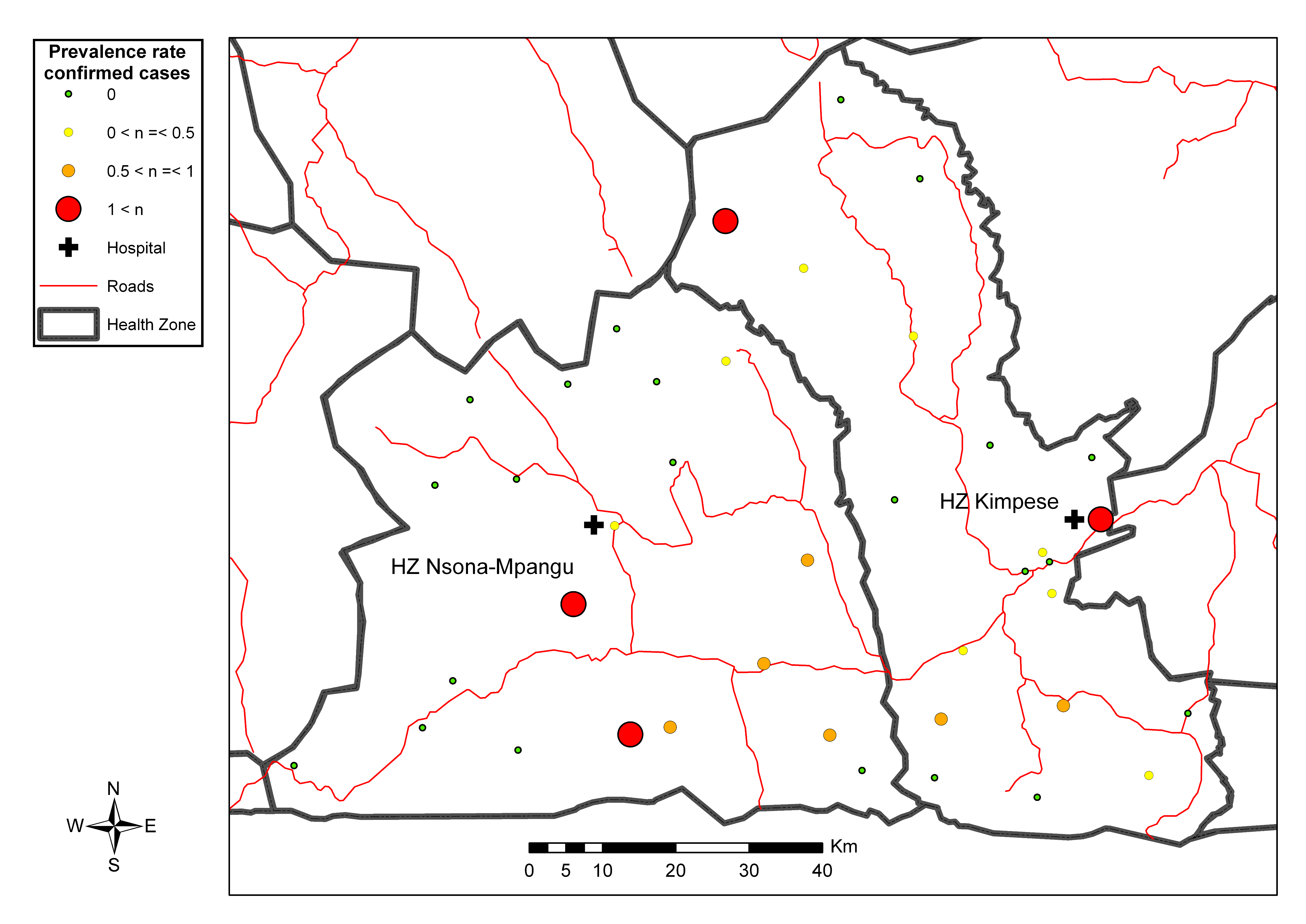

Supplement: Figure S1 — Distribution of confirmed active BU cases in the Songololo Territory, July–August 2008. (TIF) [file pntd.0002563.s002.tif]

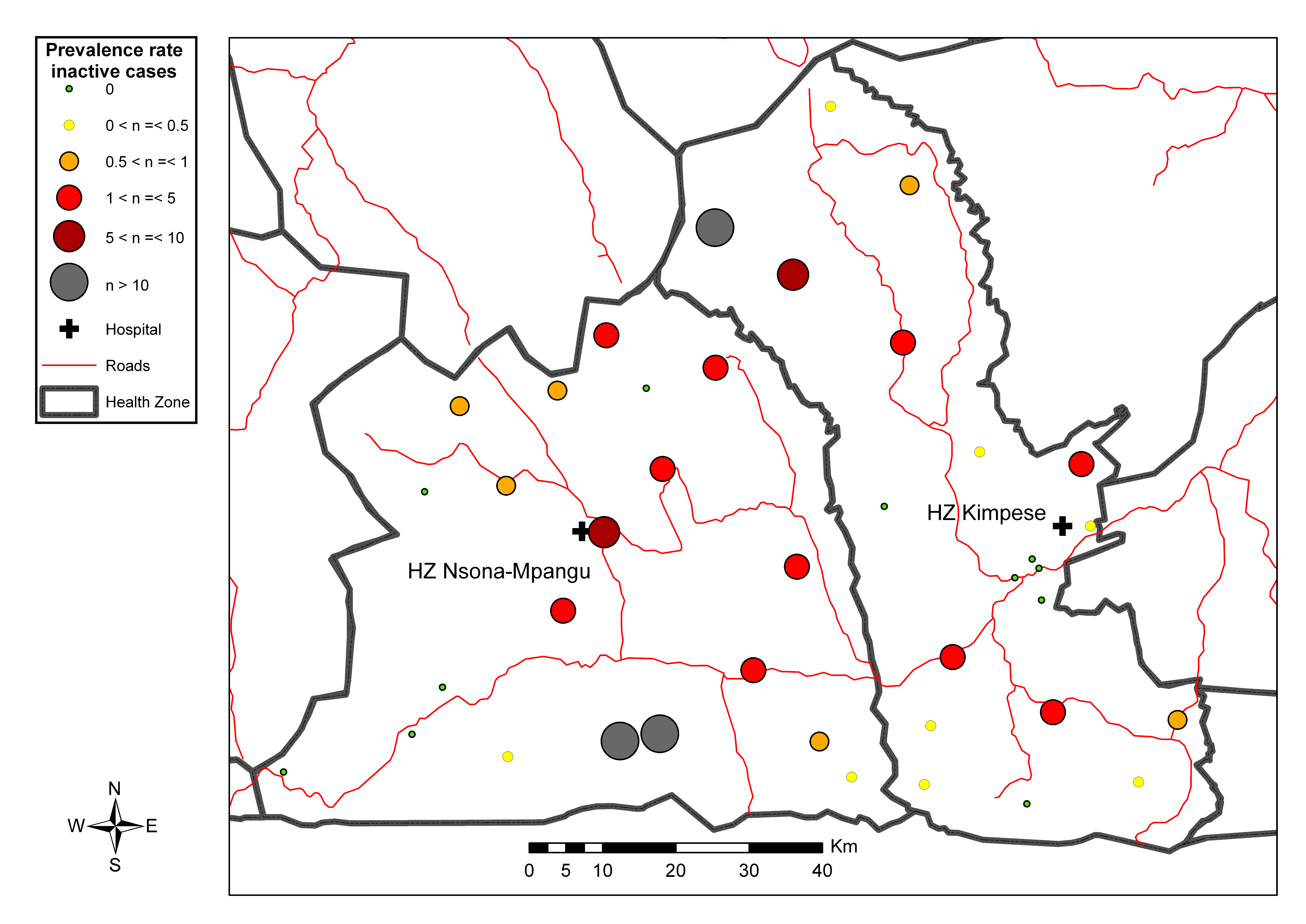

Supplement: Figure S2 — Distribution of inactive BU cases in the Songololo Territory, July–August 2008. (TIF) [file pntd.0002563.s003.tif]
