# Supplementary material for: Burden of Mycobacterium ulcerans Disease (Buruli Ulcer) and the Underreporting Ratio in the Territory of Songololo, Democratic Republic of Congo
Source: PLoS Negl Trop Dis. 2013 Dec 5;7(12):e2563. doi: 10.1371/journal.pntd.0002563 (PMC3855042; doi:10.1371/journal.pntd.0002563)
Supplement: Table S1 — Distribution of active and inactive BU cases in the Rural Health Zone of Kimpese (July–August 2008). (DOCX) [file pntd.0002563.s004.docx]

| **Health areas** | **Population 2008** | **Active cases** | | **Inactive cases** | | **Total** | |
| --- | --- | --- | --- | --- | --- | --- | --- |
|  |  |  |  |  |  |  |  |
|  |  | Number | Prevalence (x1000) | Number | Prevalence (x1000) | Number | Prevalence (x1000) |
| **BEMBA** | 3220 | 2 | 0,6 | 2 | 0,6 | 4 | 1,2 |
| **CBCO** | 13046 | 3 | 0,2 | 0 | 0,0 | 3 | 0,2 |
| **CECO** | 9948 | 3 | 0,3 | 0 | 0,0 | 3 | 0,3 |
| **KASI** | 11413 | 25 | 2,2 | 77 | 6,7 | 102 | 8,9 |
| **KIASUNGUA** | 6349 | 1 | 0,2 | 1 | 0,2 | 2 | 0,3 |
| **KIKEBA** | 4872 | 5 | 1,0 | 1 | 0,2 | 6 | 1,2 |
| **KILUEKA** | 5382 | 2 | 0,4 | 8 | 1,5 | 10 | 1,9 |
| **KIMBALA** | 11478 | 18 | 1,6 | 1 | 0,1 | 19 | 1,7 |
| **KIMBANGUISTE** | 16180 | 2 | 0,1 | 0 | 0,0 | 2 | 0,1 |
| **LOVO** | 4893 | 7 | 1,4 | 8 | 1,6 | 15 | 3,1 |
| **LUKUNGA** | 5201 | 6 | 1,2 | 3 | 0,6 | 9 | 1,7 |
| **MALANGA** | 4170 | 3 | 0,7 | 0 | 0,0 | 3 | 0,7 |
| **MBANZA NSANDA** | 4381 | 5 | 1,1 | 1 | 0,2 | 6 | 1,4 |
| **MUKIMBUNGU** | 5070 | 29 | 5,7 | 95 | 18,7 | 124 | 24,5 |
| **NKUANZA** | 9628 | 7 | 0,7 | 31 | 3,2 | 38 | 3,9 |
| **SONGA LUMUENO** | 6919 | 2 | 0,3 | 0 | 0,0 | 2 | 0,3 |
| **VIAZA** | 3520 | 7 | 2,0 | 5 | 1,4 | 12 | 3,4 |
| **VILA** | 5309 | 4 | 0,8 | 1 | 0,2 | 5 | 0,9 |
| **VUNDA NSOLE** | 3336 | 2 | 0,6 | 1 | 0,3 | 3 | 0,9 |
| **YANGA DIA SONGA** | 11793 | 8 | 0,7 | 0 | 0,0 | 8 | 0,7 |
| **Total** | **146108** | **141** | **1,0** | **235** | **1,6** | **376** | **2,6** |

**Supporting Table S1: Distribution of active and inactive BU cases in the Rural Health Zone of Kimpese (July-August 2008)**
