# Supplementary material for: Burden of Mycobacterium ulcerans Disease (Buruli Ulcer) and the Underreporting Ratio in the Territory of Songololo, Democratic Republic of Congo
Source: PLoS Negl Trop Dis. 2013 Dec 5;7(12):e2563. doi: 10.1371/journal.pntd.0002563 (PMC3855042; doi:10.1371/journal.pntd.0002563)
Supplement: Table S2 — Distribution of active and inactive BU cases in the Rural Health Zone of Nsona Mpangu (July–August 2008). (DOCX) [file pntd.0002563.s005.docx]

| **Health areas** | **Population 2008** | **Active cases** | | **Inactive cases** | | **Total** | |
| --- | --- | --- | --- | --- | --- | --- | --- |
|  |  |  |  |  |  |  |  |
|  |  | Number | Prevalence (x1000) | Number | Prevalence (x1000) | Number | Prevalence (x1000) |
| **KINGANGA** | 4714 | 1 | 0,2 | 3 | 0,6 | 4 | 0,8 |
| **KINZOLANI** | 1991 | 1 | 0,5 | 9 | 4,5 | 10 | 5,0 |
| **KISONGA** | 3380 | 13 | 3,8 | 80 | 23,7 | 93 | 27,5 |
| **KIZULU** | 5425 | 2 | 0,4 | 1 | 0,2 | 3 | 0,6 |
| **LOMBE** | 3829 | 6 | 1,6 | 3 | 0,8 | 9 | 2,4 |
| **LUANIKA** | 3062 | 2 | 0,7 | 1 | 0,3 | 3 | 1,0 |
| **LUFU** | 4728 | 14 | 3,0 | 17 | 3,6 | 31 | 6,6 |
| **MANTEKE** | 3355 | 1 | 0,3 | 2 | 0,6 | 3 | 0,9 |
| **MAYANGA** | 5667 | 4 | 0,7 | 9 | 1,6 | 13 | 2,3 |
| **MBANZA NGOMBE** | 2206 | 3 | 1,4 | 0 | 0,0 | 3 | 1,4 |
| **MBANZA NKAZI** | 3474 | 1 | 0,3 | 2 | 0,6 | 3 | 0,9 |
| **MINKELO** | 5428 | 9 | 1,7 | 21 | 3,9 | 30 | 5,5 |
| **NDUIZI** | 4162 | 0 | 0,0 | 0 | 0,0 | 0 | 0,0 |
| **NGOMBE** | 8582 | 1 | 0,1 | 0 | 0,0 | 1 | 0,1 |
| **NKAMUNA** | 4689 | 17 | 3,6 | 59 | 12,6 | 76 | 16,2 |
| **NKENGE** | 7759 | 0 | 0,0 | 0 | 0,0 | 0 | 0,0 |
| **NSONA MPANGU** | 3774 | 4 | 1,1 | 24 | 6,4 | 28 | 7,4 |
| **NTADI A NZADI** | 2527 | 6 | 2,4 | 12 | 4,7 | 18 | 7,1 |
| **PALABALA** | 2451 | 0 | 0,0 | 0 | 0,0 | 0 | 0,0 |
| **SONGOLOLO** | 10107 | 33 | 3,3 | 38 | 3,8 | 71 | 7,0 |
| **Total** | **91310** | **118** | **1,3** | **281** | **3,1** | **399** | **4,4** |

Supporting Table S2: Distribution of active and inactive BU cases in the Rural Health Zone of Nsona Mpangu (July-August 2008).
