# Supplementary material for: Burden of Mycobacterium ulcerans Disease (Buruli Ulcer) and the Underreporting Ratio in the Territory of Songololo, Democratic Republic of Congo
Source: PLoS Negl Trop Dis. 2013 Dec 5;7(12):e2563. doi: 10.1371/journal.pntd.0002563 (PMC3855042; doi:10.1371/journal.pntd.0002563)
Supplement: Table S3 — Comparison of active case features in the two Rural Health Zones of Songololo Territory, July–August 2008. (DOCX) [file pntd.0002563.s006.docx]

| **Supporting Table S3. Comparison of active case features in the two Rural Health Zones of Songololo Territory, July-August 2008.** | | | | | | | | | | | | |  |
| --- | --- | --- | --- | --- | --- | --- | --- | --- | --- | --- | --- | --- | --- |
|  | |  |  | |  |  | | | |  |  |  | |
| **Characteristic** | |  | **RHZ Kimpese (n=141)** | | **RHZ Nsona-Mpangu (n=118)** | | **p-value** |  |  |  |  |  |  |
|  | |  | **n (%)** | | **n (%)** | |  |  |  |  |  |  |  |
| Gender | | Female | | 66 (46.8) | 64 (54.2) | | 0.234 |  |  |  |  |  |  |
|  | | Male | | 75 (53.2) | 54 (45.8) | |  |  |  |  |  |  |  |
| Age | | ≤ 15 years | | 45 (31.9) | 43 (36.4) | | 0.332 |  |  |  |  |  |  |
|  | | 16-49 years | | 63 (44.7) | 56 (47.5) | |  |  |  |  |  |  |  |
|  | | > 49 years | | 33 (23.4) | 19 (16.1) | |  |  |  |  |  |  |  |
|  |  |  |  |  |  |  |  |  |  |  |  |  |  |
| Classification of cases | | New case | | 133 (94.3) | 110 (93.2) | | 0.712 |  |  |  |  |  |  |
|  | | Relapse | | 8 (5.7) | 8 (6.8) | |  |  |  |  |  |  |  |
|  |  |  |  |  |  |  |  |  |  |  |  |  |  |
| Clinical forms | | Ulcerated simple | | 104 (73.8) | 74 (62.7) | | <0.001 |  |  |  |  |  |  |
|  | | Ulcerated mixed | | 13 (9.2) | 1 (0.8) | |  |  |  |  |  |  |  |
|  | | Non ulcerated | | 24 (17.0) | 43 (36.4) | |  |  |  |  |  |  |  |
| Category of lesion | | I | | 56 (41.2)† | 68 (57.6) | | 0.031 |  |  |  |  |  |  |
|  | | II | | 50 (36.8)† | 30 (25.4) | |  |  |  |  |  |  |  |
|  | | III | | 30 (22.1)† | 20 (16.9) | |  |  |  |  |  |  |  |
| Functional limitation | | Yes | | 50 (35.5) | 12 (10.2) | | <0.001 |  |  |  |  |  |  |
|  | | No | | 91 (64.5) | 106 (89.8) | |  |  |  |  |  |  |  |
| Site of lesion | | Lower limb | | 96 (68.1) | 79 (66.4)‡ | | 0.896 |  |  |  |  |  |  |
|  | | Upper limb | | 31 (22.0) | 29 (24.4)‡ | |  |  |  |  |  |  |  |
|  | | Other | | 14 (9.9) | 11 (9.2)‡ | |  |  |  |  |  |  |  |
| Confirmation | | Yes | | 41 (29.1) | 31 (26.3) | | 0.615 |  |  |  |  |  |  |
|  | | No | | 100 (70.9) | 87 (73.7) |  | | | |  |  |  | |
|  | |  |  | |  |  | | | |  |  |  | |
|  | |  | | | |  | | | |  |  |  | |
|  | |  |  | |  |  | | | |  |  |  | |

† n=136 because of 5 missing data

‡ n=119 because of one case with disseminated lesions
