# Supplementary material for: Burden of Mycobacterium ulcerans Disease (Buruli Ulcer) and the Underreporting Ratio in the Territory of Songololo, Democratic Republic of Congo
Source: PLoS Negl Trop Dis. 2013 Dec 5;7(12):e2563. doi: 10.1371/journal.pntd.0002563 (PMC3855042; doi:10.1371/journal.pntd.0002563)
Supplement: Table S4 — Comparison of inactive case features in the two Rural Health Zones of Songololo Territory, July–August 2008. (DOCX) [file pntd.0002563.s007.docx]

| **Supporting Table S4. Comparison of inactive case features in the two Rural Health Zones of Songololo Territory, July-August 2008.** | | | | | | | | | | | | | |
| --- | --- | --- | --- | --- | --- | --- | --- | --- | --- | --- | --- | --- | --- |
|  | |  | |  | |  |  | | | |  |  |  |
| **Characteristic** |  | | **RHZ Kimpese (n=235)** | | **RHZ Nsona-Mpangu (n=281)** | | | **p-value** |  |  |  |  |  |
|  |  | | **n (%)** | | **n (%)** | | |  |  |  |  |  |  |
| Gender | Female | | 156 (66.4) | | 179 (63.7) | | | 0.524 |  |  |  |  |  |
|  | Male | | 79 (33.6) | | 102 (36.3) | | |  |  |  |  |  |  |
| Age | ≤ 15 years | | 67 (28.5) | | 69 (24.5) | | | 0.552 |  |  |  |  |  |
|  | 16-49 years | | 123 (52.3) | | 159 (56.6) | | |  |  |  |  |  |  |
|  | > 49 years | | 45 (19.1) | | 53 (18.9) | | |  |  |  |  |  |  |
| Functional limitation | Yes | | 17 (7.2) | | 6 (2.1) | | | 0.005 |  |  |  |  |  |
|  | No | | 218 (92.8) | | 275 (97.9) | | |  |  |  |  |  |  |
| Site of scar | Lower limb | | 135 (54.4)† | | 168 (57.3)‡ | | | 0.788 |  |  |  |  |  |
|  | Upper limb | | 76 (30.6)† | | 85 (29.0)‡ | | |  |  |  |  |  |  |
|  | Other | | 37(15.0 )† | | 40 (13.7)‡ | | |  |  |  |  |  |  |
|  | |  | | | | |  | | | |  |  |  |
|  | |  | |  | |  |  | | | |  |  |  |

†n=248 because of patients with disseminated lesions

‡n=293 because of patients with disseminated lesions
